# Genito-Urinary Surgery

**Published:** 1904-12-24

**Authors:** 


### GENITO-URINARY SURGERY.

**Renal Decapsulation for Chronic Bright's Disease.**—George M. Edebohls,[1] who introduced the above procedure, remains as enthusiastic a supporter of the treatment as ever. In his last communication on the subject he states that he would feel it a dereliction of duty not to advise the operation, if three conditions are fulfilled and if the patient has a reasonable expectation of not less than a month of life without operation. The three conditions are: first, the clear establishment of the diagnosis of chronic Bright's disease; secondly, the absence in the given case of absolute contra-indications to any operation; and, thirdly, the possibility of securing the service of a surgeon reasonably familiar with practical renal surgery. The symptoms which most definitely indicate impending dissolution in chronic Bright's disease, the author considers to be the dyspnœa or air hunger of the final stage, whether due to lung involvement, dilatation of the heart or poisoning of the respiratory centres. The author has a number of times found that, at periods remote from the operation, cardiac hypertrophies and derangements, existing before operation, had become insignificant or had disappeared as a result of the operation. In deciding for or against operation the most important point is to discover whether the degree of cardiac compensation is sufficiently good to warrant the assumption that the patient will safely stand the anæsthesia. This turns on whether the enlargement of the heart is mainly hypertrophic or mainly due to dilatation. Albuminuric retinitis is of most serious import. Sixty-seven per cent. of patients with retinitis will die within a year and probably none will survive two years. Edebohls operated in nine cases with retinitis and all the patients died within a year. In successful cases the benefits of the operation are shown by the return of colour and strength, the disappearance of dropsies, headaches, and backaches, and improvement in the condition and action of the heart. The author advises against fixing the kidneys as well as decapsulating them, unless there are special symptoms due to mobility of the kidney. The operation has not had yet a full and fair trial, for, as a rule, only severe and advanced cases have to the present time been subjected to it.

**Unsuspected Lesions in Movable Kidneys.**—Frank E. Taylor[2] reports five cases of movable kidney in which, at the operation for fixation, lesions were found in the kidneys which had not been previously suspected. In two of the cases renal calculus was found; in two, tubercular disease, necessitating partial or complete nephrectomy, and in the fifth case tnere was hydro-nephrosis and congenital smallness of the ureter. The consideration of these cases shows that a movable kidney is not uncommonly the seat of disease which is unsuspected before exploration. The cases show also the advisability of bringing movable kidneys out upon the loin when operating and of thoroughly examining them.

**Urinary Tuberculosis.**—Hunner of Baltimore[3] has dealt with this subject in a paper in which 35 cases occurring in women are related. The cases demonstrate the frequency of primary renal tuberculosis and the rarity of primary vesical tuberculosis. The kidney disease frequently causes bladder symptoms, especially pain and frequency of micturition, when the bladder is free from disease. Further, renal tuberculosis is often unilateral. For these reasons early treatment gives good results. The importance of cystoscopy in diagnosis is insisted upon.

[1] Abst. Nova Medica, July, 1904, p. 2. [2] Annals of Surgery, August, 1904, p. 215. [3] Abst. Scottish Med. and Surg. Jour., August, 1904, p. 181.

*(To be continued.)*

MINER'S PHTHISIS.—An inquiry into efficacious means of allaying stone dust in mines, which was recently held by the Institution of Mining and Metallurgy, shows how very difficult it is to find a reliable remedy. The recommendations following the inquiry were in favour of the use of an apparatus known as James's water blast. This blast should be used to damp the stone before operations are begun, and its general adoption would probably do much to reduce the prevalence of miner's phthisis.

